# Supplementary figures and images for: Reliability and validity of the Forgotten Joint Score-12 for total ankle replacement and ankle arthrodesis
Source: PLoS One. 2023 Jun 14;18(6):e0286762. doi: 10.1371/journal.pone.0286762 (PMC10266669; doi:10.1371/journal.pone.0286762)

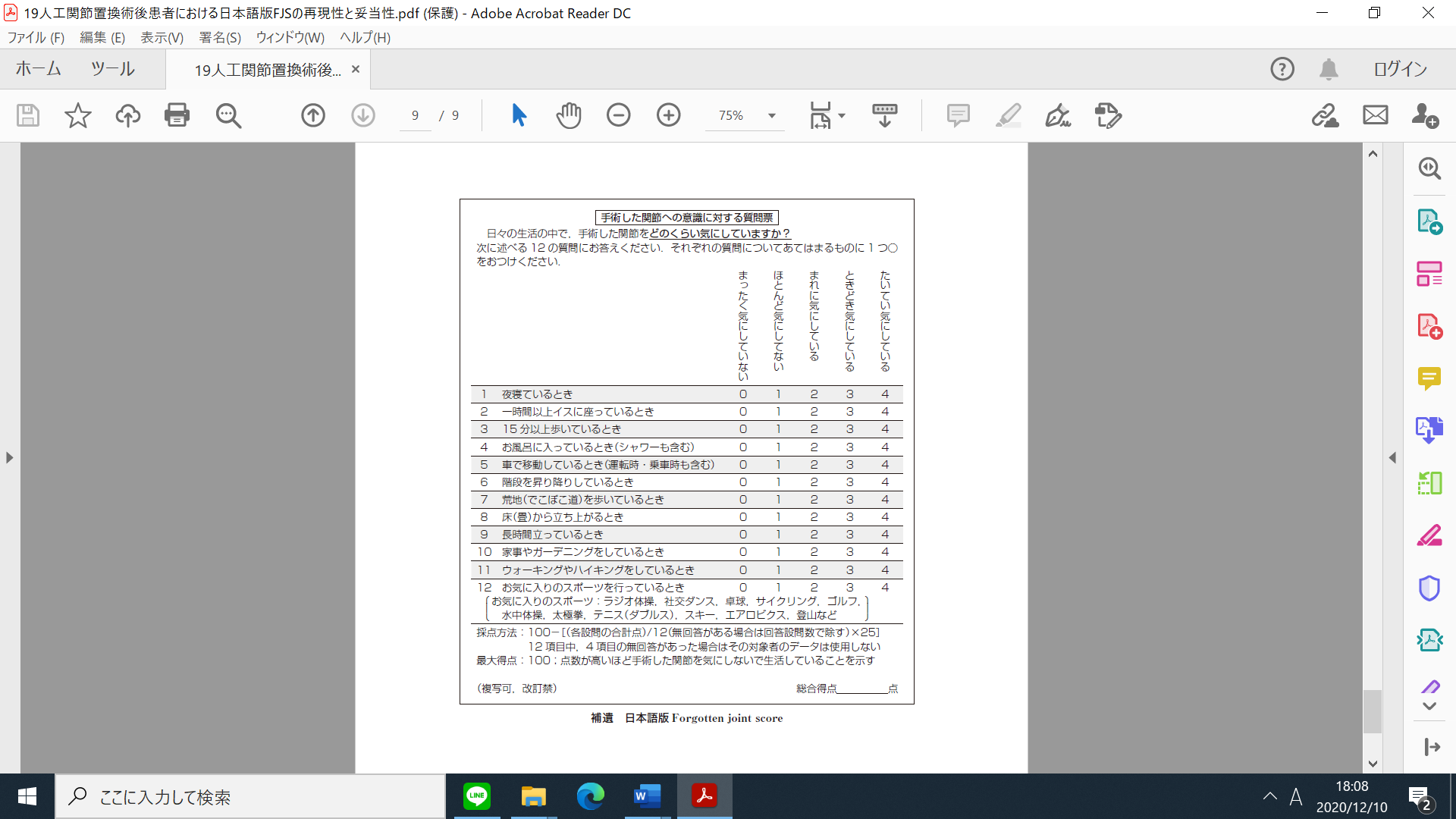

Supplement: S1 File — (DOCX) [file pone.0286762.s001.docx]
