# Supplementary material for: Reliability and validity of the Forgotten Joint Score-12 for total ankle replacement and ankle arthrodesis
Source: PLoS One. 2023 Jun 14;18(6):e0286762. doi: 10.1371/journal.pone.0286762 (PMC10266669; doi:10.1371/journal.pone.0286762)
Supplement: Attachment — Submitted filename: Response to Reviewers.docx [file pone.0286762.s003.docx]

[Editor comment]

1. Please ensure that your manuscript meets PLOS ONE’s style requirements, including those for file naming. The PLOS ONE style templates can be found at 
<https://journals.plos.org/plosone/s/file?id=wjVg/PLOSOne_formatting_sample_main_body.pdf> and 
<https://journals.plos.org/plosone/s/file?id=ba62/PLOSOne_formatting_sample_title_authors_affiliations.pdf>

[Author response]

We confirmed that the revised manuscript met the PLOS ONE’s style requirements**.**

[Editor comment]

2. In your Data Availability statement, you have not specified where the minimal data set underlying the results described in your manuscript can be found. PLOS defines a study’s minimal data set as the underlying data used to reach the conclusions drawn in the manuscript and any additional data required to replicate the reported study findings in their entirety. All PLOS journals require that the minimal data set be made fully available. For more information about our data policy, please see <http://journals.plos.org/plosone/s/data-availability.>

We will update your Data Availability statement to reflect the information you provide in your cover letter.

[Author response]

**We added the** Supporting Information to the revised manuscript. We also provided the Data Availability statement in the cover letter

[Author action]

S1 and S2 were submitted to the revised manuscript and mentioned in the main text as follows.

“The Japanese version was created using a cross-cultural translation and adaptation process (S1)” (L81 of the revised manuscript)

“... and the MDC^95^ was 18.0 (S2).” (L247 of the revised manuscript)

“... and the MDC^95^ value was 7.2 (S2).” (L249 of the revised manuscript)

**We provided the following statement in the cover letter.**

**“Data Availability:** All relevant data are within the paper and its Supporting Information files.”

Reviewer #1:
[Reviewer comment]

this is an interesting study and should have been conducted earlier, this will help the F&A society to improve outcome measurements.

[Author response]

We appreciate the reviewer’s comment. All comments were incorporated into the revised manuscripts. We hope it will be acceptable for publication in Plos One.

[Reviewer comment]

English could be shortened

[Author response]

According to the reviewer’s comment, redundant sentences were deleted. Furthermore, the entire manuscript was edited by an English editing expert. The word count of the manuscript was reduced from 3266 to 2877.

[Author action]

The following sentences and phrases were deleted.

“Different methods, such as clinical assessment, implant survivorship, and radiographic measurements, are available for the postoperative evaluation of TAR and AA” (L64 of the first submission)

“Although computerized adaptive tests have become common practice and can reduce the burden on patients…” (L74 of the first submission)

“…when applied after hip and knee arthroplasties” (L82 of the first submission)

“Considering the widespread use in patients with knee and hip diseases, it will also be widely used in patients with foot and ankle diseases, such as ankle arthritis.” (L92 of the first submission)

“…such as being asleep, climbing stairs, taking a bath, standing for long periods, and sports…” (L138 of the first submission)

“Patients answered each item on a 5-point Likert scale, except for Question 3, which was answered using a pain visual analog scale (VAS).” (L148 of the first submission)

“…(2) much improved, (3) slightly improved, (4) unchanged, (5) slightly worse, (6) much worse…” (L163 of the first submission)

“MDC is the minimal change that falls outside the measurement error in the score of an instrument. “ (L195 of the first submission)

“…with a correlation coefficient of 0.70 for the descriptive system index value and 0.51 for the EQ VAS” (L245 of the first submission)

“Our results agree with the hypothesis that the correlation of FJS-12 with the ankle disease-specific PROMs (i.e., SAFE-Q) was high, while the correlation of FJS-12 with the general health PROMs (EQ-5D-5L) was relatively low.” (L284 of the first submission)

“Furthermore, the patients who underwent hip arthroscopy had varying degrees of awareness regarding their operated joints.” (L309 of the first submission)

“…because younger patients, such as those with hemophilic ankle arthritis, can also undergo TAR and AA.” (L324 of the first submission)

“Therefore, it is adequate as a comparator for the Japanese FJS-12.” (L334 of the first submission)

“The Japanese FJS-12 was created through a cross-cultural translation and adaptation process.” (L335 of the first submission)

We made corrections in the following lines.

L 32, 33, 34, 37, 38, 40, 43-45, 47, 48, 51-53, 55, 57, 58, 61, 63, 65, 69-712, 75, 76, 78, 79, 8-83, 85-87, 90, 92, 98-100, 114, 115, 118, 119, 121, 123, 127-129, 131, 132, 136-139, 141-144, 147-149, 154-158, 160, 162, 163, 165-170, 176, 177, 189-191, 199, 215, 217-220, 222, 229, 232-234, 239, 243, 244, 251, 253, 258, 262, 264, 268, 273, 274, 279, 281-283, 285-288, 291, 292, 294-298, 300-307, 312-316, 318, 321.

[Reviewer comment]

Spelling mistakes on page 12 line 201 and 202

[Author response]

We apologize for the mistakes. The word “INsruments” were corrected.

We capitalized several words according to the original term, COSMIN (COnsensus-based Standards for the selection of health Measurement Instruments, <https://www.cosmin.nl/>). Therefore, we did not make changes.

[Author action]

The corrections were made as follows.

“...the COnsensus-based Standards for the selection of health Measurement INstruments...“ (L189 of the revised manuscript)

[Reviewer comment]

Why did you not statistic testing on patients groups (Tbl1)

[Author response]

According to the reviewer’s comment, we added the results of statistic testing to Table 1. The statistical methods were addressed in the Methods. The results were added to the Results.

As already stated in the Methods, we analyzed the TAR and AA groups separately because comparing the two groups was not the purpose of this study. Moreover, we do not believe comparing the TAR and AA groups provides meaningful information because the indications of the two surgeries differ. Generally, younger and more physically active patients are likely to undergo AA, while older and less active patients are likely to undergo ATR.

[Author action]

The following sentence in the Methods was deleted.

“The TAR and AA groups were analyzed separately to evaluate FJS-12 for both operations and not to compare the two operations.” (L170 of the first submission)

The following sentences were added to the Methods.

“Patients’ demographics and questionnaire results were compared between the TAR and AA groups using the [Wilcoxon signed-rank test](https://search.yahoo.co.jp/search?p=wilcoxon%20signed-rank%20test%20%E3%82%A8%E3%82%AF%E3%82%BB%E3%83%AB&rkf=1&fr=top_ga1_sa&ei=UTF-8&ts=13001&aq=2&oq=wilcoxon&at=s&ai=e8258ebe-be8a-4f41-abbd-ee64a26273fe&x=nl)s, Student t-test, and Fisher’s exact test as appropriate.” (L155 of the revised manuscript)

“To assess known-group validity, the FJS-12 scores were compared between the TAR and AA groups using the Student t-test. We hypothesized that the scores were similar between the groups.” (L168 of the revised manuscript)

We added the following sentence in the Results.

“There was no significant difference in the FJS-12 score between the two groups, although the score was 7 points higher in the TAR group than in the AA group (P = 0.20, Table 1).” (L232 of the revised manuscript)

We made the following revision.

“Among the 14 hypotheses, 13 corresponded with our hypotheses.”

→“Among the 15 hypotheses, 14 corresponded with our hypotheses.” (L234 of the revised manuscript)

Table 1 was revised as follows.

|  |  | TAR group (n = 50) | AA group (n = 65) | *P* |
| --- | --- | --- | --- | --- |
| Age (years) |  | 78 (72, 81) | 70 (62, 73) | < 0.001 |
| Sex [Women, n (%)] |  | 40 (80) | 47 (72) | 0.39 |
| Diagnosis [n (%)] | Idiopathic OA | 23 (46) | 24 (37) | 0.008 |
|  | Post-traumatic OA | 8 (16) | 13 (20) |  |
|  | Post-sprain OA | 7 (14) | 24 (37) |  |
|  | Rheumatoid arthritis | 9 (18) | 3 (5) |  |
|  | Others | 3 (6) | 1 (2) |  |
| Postoperative follow-up (months) |  | 29 (17, 56) | 34 (19, 51) | 0.48 |
| Operative procedure [n (%)] | TNK ankle | 13 (26) | n.a. |  |
|  | TNK ankle + total talar prosthesis | 17 (34) | n.a. |  |
|  | FINE Ankle | 1 (2) | n.a. |  |
|  | TM Ankle | 19 (38) | n.a. |  |
|  | Arthroscopic arthrodesis | n.a. | 60 (92) |  |
|  | Open arthrodesis | n.a. | 5 (8) |  |
| Bilateral operation [n (%)] |  | 2 (4) | 3 (5) | 1.00 |
| Revision operation [n (%)] |  | 1 (2) | 3 (5) | 0.63 |
| Interval between 1^st^ and 2^nd^ FJS (days) |  | 14 (14, 14) | 14 (14, 15) | 0.09 |
| FJS-12 (mean ± standard deviation) |  | 65 ± 20 | 58 ± 26 | 0.20 |
| SAFE-Q | Pain | 87 (77, 100) | 94 (79, 100) | 0.15 |
|  | Physical Functioning | 76 (68, 87) | 77 (64, 89) | 0.79 |
|  | Social Functioning | 88 (74, 100) | 100 (75, 100) | 0.06 |
|  | Shoe-Related | 83 (67, 100) | 83 (67, 96) | 0.27 |
|  | General Health | 93 (80, 100) | 90 (80, 100) | 0.94 |
| EQ-5D-5L | Descriptive system index value | 0.86 (0.74, 0.89) | 0.89 (0.78, 1) | 0.21 |
|  | EQ VAS | 80 (74, 90) | 80 (70, 90) | 0.63 |

Reviewer #2:

[Reviewer comment]

The manuscript aimed to examine the reliability and validity of the Japanese Forgotten Joint Score-12 (FJS-12) in patients with total ankle replacement (TAR) and ankle arthrodesis (AA). The manuscript is interesting, well-written, and presented in an organized fashion. I have only one comment on the work as presented below

[Author response]

We appreciate the reviewer’s comment. All comments were incorporated into the revised manuscripts. We hope it will be acceptable for publication in Plos One.

[Reviewer comment]

1- Abstract, please report the SEM and MDC in the Results section.

[Author response]

According to the reviewer’s comment, we reported the MDC values in the Abstract. The SEM values were not included because of the word count limit.

[Author action]

The following sentence was added to the Abstract.

“The 95% minimal detectable change values were 18.0 and 7.2 points in the TAR and AA groups, respectively.” (L51 of the revised manuscript)

[Reviewer comment]

As well, the Conclusion should consider that the Japanese version has been evaluated for metric characteristics and found reliable and valid.

[Author response]

According to the reviewer’s suggestion, we stated that the Japanese version was evaluated.

[Author action]

The Conclusion of the Abstract was revised as follows.

“The Japanese version of the FJS-12 had…” (L55 of the revised manuscript)

The Methods section of the Abstract was revised for consistency.

“The patients completed the Japanese version of the FJS-12…” (L37 of the revised manuscript)

We deleted the following phrase from the Abstract to keep the word count within the submission guidelines.

“87 women and 28 men” (L43 of the first submission)

[Reviewer comment]

2- Please use appropriate statistical test to compare the two groups of TAR and AA on the FJS-12 scores. The result may indicate the discriminant ability of the questionnaire.

[Author response]

We appreciate the thoughtful comment. According to the reviewer’s comment, we compared the FJS scores between the TAR and AA groups. This analysis was performed as a part of construct validity and hypothesis testing according to the COnsensus-based Standards for the selection of health Measurement INsruments.

However, we do not believe comparing the TAR and AA groups provides meaningful information because the indications of the two surgeries differ. Generally, younger and more physically active patients are likely to undergo AA, while older and less active patients are likely to undergo ATR.

[Author action]

The following sentence in the Methods was deleted.

“The TAR and AA groups were analyzed separately to evaluate FJS-12 for both operations and not to compare the two operations.” (L170 of the first submission)

The following sentences were added to the Methods.

“Patients’ demographics and questionnaire results were compared between the TAR and AA groups using the [Wilcoxon signed-rank test](https://search.yahoo.co.jp/search?p=wilcoxon%20signed-rank%20test%20%E3%82%A8%E3%82%AF%E3%82%BB%E3%83%AB&rkf=1&fr=top_ga1_sa&ei=UTF-8&ts=13001&aq=2&oq=wilcoxon&at=s&ai=e8258ebe-be8a-4f41-abbd-ee64a26273fe&x=nl)s, Student t-test, and Fisher’s exact test as appropriate.” (L155 of the revised manuscript)

“To assess known-group validity, the FJS-12 scores were compared between the TAR and AA groups using the Student t-test. We hypothesized that the scores were similar between the groups.” (L168 of the revised manuscript)

We added the following sentence in the Results.

“There was no significant difference in the FJS-12 score between the two groups, although the score was 7 points higher in the TAR group than in the AA group (P = 0.20, Table 1).” (L232 of the revised manuscript)

We made the following revision.

“Among the 14 hypotheses, 13 corresponded with our hypotheses.”

→“Among the 15 hypotheses, 14 corresponded with our hypotheses.” (L234 of the revised manuscript)

Table 1 was revised as follows.

|  |  | TAR group  (n = 50) | AA group  (n = 65) | *P* |
| --- | --- | --- | --- | --- |
| Age (years) |  | 78 (72, 81) | 70 (62, 73) | < 0.001 |
| Sex (Women, n [%]) |  | 40 (80) | 47 (72) | 0.39 |
| Diagnosis (n [%)]) | Idiopathic OA | 23 (46) | 24 (37) | 0.008 |
|  | Post-traumatic OA | 8 (16) | 13 (20) |  |
|  | Post-sprain OA | 7 (14) | 24 (37) |  |
|  | Rheumatoid arthritis | 9 (18) | 3 (5) |  |
|  | Others | 3 (6) | 1 (2) |  |
| Postoperative follow-up (months) |  | 29 (17, 56) | 34 (19, 51) | 0.48 |
| Operative procedure (n [%]) | TNK ankle | 13 (26) | n.a. |  |
|  | TNK ankle + total talar prosthesis | 17 (34) | n.a. |  |
|  | FINE Ankle | 1 (2) | n.a. |  |
|  | TM Ankle | 19 (38) | n.a. |  |
|  | Arthroscopic arthrodesis | n.a. | 60 (92) |  |
|  | Open arthrodesis | n.a. | 5 (8) |  |
| Bilateral operation (n [%]) |  | 2 (4) | 3 (5) | 1.00 |
| Revision operation (n [%]) |  | 1 (2) | 3 (5) | 0.63 |
| Interval between 1^st^ and 2^nd^ FJS (days) |  | 14 (14, 14) | 14 (14, 15) | 0.09 |
| FJS-12 (mean ± standard deviation) |  | 65 ± 20 | 58 ± 26 | 0.20 |
| SAFE-Q | Pain | 87 (77, 100) | 94 (79, 100) | 0.15 |
|  | Physical Functioning | 76 (68, 87) | 77 (64, 89) | 0.79 |
|  | Social Functioning | 88 (74, 100) | 100 (75, 100) | 0.06 |
|  | Shoe-Related | 83 (67, 100) | 83 (67, 96) | 0.27 |
|  | General Health | 93 (80, 100) | 90 (80, 100) | 0.94 |
| EQ-5D-5L | Descriptive system index value | 0.86 (0.74, 0.89) | 0.89 (0.78, 1) | 0.21 |
|  | EQ VAS | 80 (74, 90) | 80 (70, 90) | 0.63 |

Reviewer #3:

[Reviewer comment]

The authors have provided a purposeful and unique study. A comprhensive English editing is required to shorten the length of the sentences and also grammatical issues should be handled to improve the comprehensibility of the paper.

[Author response]

We appreciate the reviewer’s comment. Although the previous manuscript was edited by an English editing expert, the entire manuscript was again edited by another expert. Redundant sentences were also deleted. As a result, the word count of the manuscript was reduced from 3590 to 2877. We hope the revised manuscript will be acceptable for publication.

[Author action]

The following sentences and phrases were deleted.

“Different methods, such as clinical assessment, implant survivorship, and radiographic measurements, are available for the postoperative evaluation of TAR and AA” (L64 of the first submission)

“Although computerized adaptive tests have become common practice and can reduce the burden on patients…” (L74 of the first submission)

“…when applied after hip and knee arthroplasties” (L82 of the first submission)

“Considering the widespread use in patients with knee and hip diseases, it will also be widely used in patients with foot and ankle diseases, such as ankle arthritis.” (L92 of the first submission)

“…such as being asleep, climbing stairs, taking a bath, standing for long periods, and sports…” (L138 of the first submission)

“Patients answered each item on a 5-point Likert scale, except for Question 3, which was answered using a pain visual analog scale (VAS).” (L148 of the first submission)

“…(2) much improved, (3) slightly improved, (4) unchanged, (5) slightly worse, (6) much worse…” (L163 of the first submission)

“MDC is the minimal change that falls outside the measurement error in the score of an instrument. “ (L195 of the first submission)

“…with a correlation coefficient of 0.70 for the descriptive system index value and 0.51 for the EQ VAS” (L245 of the first submission)

“Our results agree with the hypothesis that the correlation of FJS-12 with the ankle disease-specific PROMs (i.e., SAFE-Q) was high, while the correlation of FJS-12 with the general health PROMs (EQ-5D-5L) was relatively low.” (L284 of the first submission)

“Furthermore, the patients who underwent hip arthroscopy had varying degrees of awareness regarding their operated joints.” (L309 of the first submission)

“…because younger patients, such as those with hemophilic ankle arthritis, can also undergo TAR and AA.” (L324 of the first submission)

“Therefore, it is adequate as a comparator for the Japanese FJS-12.” (L334 of the first submission)

“The Japanese FJS-12 was created through a cross-cultural translation and adaptation process.” (L335 of the first submission)

We made corrections in the following lines.

L 32, 33, 34, 37, 38, 40, 43-45, 47, 48, 51-53, 55, 57, 58, 61, 63, 65, 69-712, 75, 76, 78, 79, 8-83, 85-87, 90, 92, 98-100, 114, 115, 118, 119, 121, 123, 127-129, 131, 132, 136-139, 141-144, 147-149, 154-158, 160, 162, 163, 165-170, 176, 177, 189-191, 199, 215, 217-220, 222, 229, 232-234, 239, 243, 244, 251, 253, 258, 262, 264, 268, 273, 274, 279, 281-283, 285-288, 291, 292, 294-298, 300-307, 312-316, 318, 321.

[Reviewer comment]

Alsa, formula based sample size calculation is required.

[Author response]

We appreciate the important comments of the reviewer. We did not perform the priori sample size calculation based on a statistical formula. As already stated in the Methods, we determined the sample size based on the COnsensus-based Standards for the selection of health Measurement INsruments, which are widely accepted guidelines of psychometric measurement properties (stated in LXX of the revised manuscript). The number of 50 patients was also recommended by another paper (Terwee et al. Quality criteria were proposed for measurement properties of health status questionnaires. J Clin Epidemiol 2007) and used in studies assessing the validity and reliability of the FJS-12 (Shadid et al. 2016). Therefore, the number of patients in our study is acceptable even though the priori sample size calculation was not conducted.

We addressed the reviewer’s point in the limitation section. Furthermore, the result of post hoc power analysis was shown. We hope that the reviewer kindly accepts our response.

[Author action]

We added the following sentences in the limitation section.

“Finally, the number of patients was not based on the sample size calculation. However, we determined the sample size according to the recommendation of the widely accepted guidelines [23]. Furthermore, the post hoc statistical power analysis using the correlation between the FJS-12 and SAFE-Q scores showed the statistical power ranged from 0.80 to 0.99.” (L312 of the revised manuscript)

The following correction was made accordingly.

“Fifth, we used the Japanese version...” (L310 of the revised manuscript)

[Other corrections]

The brackets in Table 1 were corrected.

|  |  | TAR group  (n = 50) | AA group  (n = 65) | *P* |
| --- | --- | --- | --- | --- |
| Age (years) |  | 78 (72, 81) | 70 (62, 73) | < 0.001 |
| Sex (Women, n [%]) |  | 40 (80) | 47 (72) | 0.39 |
| Diagnosis (n [%)]) | Idiopathic OA | 23 (46) | 24 (37) | 0.008 |
|  | Post-traumatic OA | 8 (16) | 13 (20) |  |
|  | Post-sprain OA | 7 (14) | 24 (37) |  |
|  | Rheumatoid arthritis | 9 (18) | 3 (5) |  |
|  | Others | 3 (6) | 1 (2) |  |
| Postoperative follow-up (months) |  | 29 (17, 56) | 34 (19, 51) | 0.48 |
| Operative procedure (n [%]) | TNK ankle | 13 (26) | n.a. |  |
|  | TNK ankle + total talar prosthesis | 17 (34) | n.a. |  |
|  | FINE Ankle | 1 (2) | n.a. |  |
|  | TM Ankle | 19 (38) | n.a. |  |
|  | Arthroscopic arthrodesis | n.a. | 60 (92) |  |
|  | Open arthrodesis | n.a. | 5 (8) |  |
| Bilateral operation (n [%]) |  | 2 (4) | 3 (5) | 1.00 |
| Revision operation (n [%]) |  | 1 (2) | 3 (5) | 0.63 |
| Interval between 1^st^ and 2^nd^ FJS (days) |  | 14 (14, 14) | 14 (14, 15) | 0.09 |
| FJS-12 (mean ± standard deviation) |  | 64 ± 20 | 58 ± 26 | 0.20 |
| SAFE-Q | Pain | 87 (77, 100) | 94 (79, 100) | 0.15 |
|  | Physical Functioning | 76 (68, 87) | 77 (64, 89) | 0.79 |
|  | Social Functioning | 88 (74, 100) | 100 (75, 100) | 0.06 |
|  | Shoe-Related | 83 (67, 100) | 83 (67, 96) | 0.27 |
|  | General Health | 93 (80, 100) | 90 (80, 100) | 0.94 |
| EQ-5D-5L | Descriptive system index value | 0.86 (0.74, 0.89) | 0.89 (0.78, 1) | 0.21 |
|  | EQ VAS | 80 (74, 90) | 80 (70, 90) | 0.63 |

*P* values in Table 2 were revised as follows.

|  |  | TAR group (n = 50) |  | AA group (n = 65) |  |
| --- | --- | --- | --- | --- | --- |
|  |  | *ρ* | *P* | *ρ* | *P* |
| SAFE-Q | Pain | 0.71 | < 0.001 | 0.62 | < 0.001 |
|  | Physical functioning | 0.61 | < 0.001 | 0.79 | < 0.001 |
|  | Social functioning | 0.39 | 0.006 | 0.71 | < 0.001 |
|  | Shoe-related | 0.61 | < 0.001 | 0.55 | < 0.001 |
|  | General health | 0.59 | < 0.001 | 0.68 | < 0.001 |
| EQ-5D-5L | Descriptive system index value | 0.34 | 0.01 | 0.70 | < 0.001 |
|  | EQ VAS | 0.05 | 0.73 | 0.51 | < 0.001 |
