# Supplementary material for: Reliability and validity of the Forgotten Joint Score-12 for total ankle replacement and ankle arthrodesis
Source: PLoS One. 2023 Jun 14;18(6):e0286762. doi: 10.1371/journal.pone.0286762 (PMC10266669; doi:10.1371/journal.pone.0286762)
Supplement: Attachment — Submitted filename: Response to Reviewers.docx [file pone.0286762.s004.docx]

Reviewer #2:

[Reviewer comment]

I appreciate the efforts of the authors in revising the manuscript. The manuscript looks better, but there are still issues that is required to be addressed.

[Author response]

We appreciate the reviewer’s comment. All issues were addressed in the revised manuscript. We hope that it will be acceptable for publication.


[Reviewer comment]

Abstract

1- Results, please report the results on know-group validity.

[Author response]

According to the reviewer’s comment, we reported the results of known-group validity.

[Author action]

The sentence in the Abstract was revised as follows.

“The mean FJS-12 scores were 65 and 58 for the TAR and AA groups, respectively, with no significant difference between groups (*P* = 0.20).” (L45 of the manuscript R2)

[Reviewer comment]

2- Conclusions, lines 55-57 are repetitions with results. I would suggest omitting them.

[Author response]

We deleted the sentences in L55-57 according to the reviewer’s comment.

[Author action]

The following sentences were deleted.

“The Japanese version of FJS-12 had a sufficient construct validity and internal consistency. It also had acceptable test-retest reliability and measurement errors. Furthermore, no floor or ceiling effects were observed.” (L55-57 of the manuscript R1)

We added the following sentence.

“The FJS-12 can be a useful tool for the postoperative assessment of patients with end-stage ankle arthritis.” (L56 of the manuscript R2)

[Reviewer comment]

Introduction
1- Page 6, line 87, “I would suggest omitting “effectiveness” and inserting “usefulness”, please.

[Author response]

According to the reviewer’s comment, effectiveness was rephrased to usefulness.

[Author action]

The sentence was revised as follows.

“Although the usefulness of FJS-12 has not…” (L86 of the manuscript R2)

[Reviewer comment]
Methods
1- Page 10, line 171, please clarify the statement on the confirmation of 75% of hypotheses for construct validity in the context of your study.

[Author response]

According to the reviewer’s comment, we added the specific numbers of the hypotheses in this study.

[Author action]

The following revision was made.

“If at least 75% of the hypotheses (12 out of 15 hypotheses) were confirmed,…” (L170 of the manuscript R2)

[Reviewer comment]

2- Page 10, line 172, please analyze the exploratory factor analysis considering all the data on Japanese FJS-12 from both groups (n=115) .

[Author response]

According to the reviewer’s comment, we performed factor analysis. The analysis was addressed in the Methods, Results, and Discussion. Due to the word count limit, we did not mention the results in the Abstract.

[Author action]

The following sentence was added to the Methods.

“**Factor analysis**

Principal component analysis with Varimax rotation was performed to assess the dimensionality of the FJS-12 using the overall data of the TAR and AA groups.” (L175 of the manuscript R2)

We added the following sentences, figure, and table to the Results.

“**Factor analysis**

The first factor explained 58.9% of the variance, with an eigenvalue of 7.06 (Fig. 3). The first factor mainly represented difficulties during activities, such as items 3, 6-8, 11, and 12 (Table 3). The second factor explained 9.9% of the variance, with an eigenvalue of 1.19. This factor mainly represented difficulties at rest, such as items 1, 2, 4, and 5.” (L249 of the manuscript R2)

“**Fig. 3. Scree plot of FJS-12.**” (L255 of the manuscript R2)

**“****Table 3. Factor loading of the FJS-12.** (L257 of the manuscript R2)

| FJS 12 items | Factor 1 | Factor 2 |
| --- | --- | --- |
| 1 | 0.249 | **0.680** |
| 2 | 0.237 | **0.872** |
| 3 | **0.599** | **0.521** |
| 4 | **0.418** | **0.604** |
| 5 | 0.385 | **0.622** |
| 6 | **0.736** | 0.212 |
| 7 | **0.830** | 0.295 |
| 8 | **0.735** | 0.294 |
| 9 | **0.733** | 0.348 |
| 10 | **0.665** | 0.329 |
| 11 | **0.720** | **0.457** |
| 12 | **0.662** | 0.350 |

Bold numbers indicate the factor loading > 0.4.

The following paragraph was added to the Discussion.

“In this study, the principal component analysis showed the two dimensions of the FJS-12, although the eigenvalue for the first factor was six times larger than that of the second factor (7.06 versus 1.09). In contrast, studies using patients after total knee arthroplasty and anterior cruciate reconstruction reported unidimensionality of the FJS-12 [24, 28]. The differences in the patient background and analysis methods are possible explanations for the discrepancy. Further studies are required to confirm the factor structure of the FJS-12 for patients after TAR and AA.” (L289 of the manuscript R2)

We added the following references.

28.	Hamilton DF, Loth FL, Giesinger JM, Giesinger K, MacDonald DJ, Patton JT, et al. Validation of the English language Forgotten Joint Score-12 as an outcome measure for total hip and knee arthroplasty in a British population. Bone Joint J. 2017;99-B:218-224. (L431 of the manuscript R2)

The reference numbers in the text and reference list were changed accordingly. (L300, 306, 307, 308, 331, 435, 439, 441, 445, 449, 453, 455 of the manuscript R2)

[Reviewer comment]

3- Page 10, line 177, on ICC, Please clarify, Single measure or average measure? Further, absolute or consistency? ICC agreement was used? Please also provide reason/reasons for using the two-way mixed-effects model.

[Author response]

We apologize for our mistake. We calculated the ICC for the two-way random-effect model, not the two-way mixed-effects model. Also, we clarified the details of the ICC.

[Author action]

The sentence was revised as follows.

“…by calculating the intraclass correlation coefficient (ICC)_agreement_ for a two-way random-effects model, single measurement. (L180 of the manuscript R2)

[Reviewer comment]

4- Page 11, line 191, how 63 patients were calculated for sample size? Terwee et al (2007) suggest at least 100 patients. This needs clarification.

[Author response]

We appreciate the reviewer’s comment. We have already stated the rationale of sample size calculation as follows.

“The sample size was determined based on the recommendation of the COnsensus-based Standards for the selection of health Measurement INstruments, in which 50 or more patients were adequate in assessing the measurement property of a PROM [23]. Assuming that 20% of patients were excluded from the analysis, we determined a sample size of 63 patients for each group.” (L188-192 of the manuscript R1).

According to COSMIN, 50 or more patients are adequate to test construct validity, internal consistency, measurement error and reliability, and known-group validity. Additionally, Terwee et al. (2007) stated that at least 50 patients are needed to test construct validity. Therefore, 63 patients would be sufficient.

[Author action]

The following revision was made. We hope that the reviewer will accept our decision.

“...we determined a sample size of 63 patients for each group (50/0.8 = 62.5).” (L195 of the manuscript R2)

[Reviewer comment]

Results
1- Page 12, Table 1, last coloumn, please report the tests used along with p values.

[Author response]

According to the reviewer’s comment, we reported the statistical tests used in the table caption.

[Author action]

Table 1 was revised as follows. (L210 of the manuscript R2)

|  |  | TAR group  (n = 50) | AA group  (n = 65) | *P* |
| --- | --- | --- | --- | --- |
| Age (years) |  | 78 (72, 81) | 70 (62, 73) | < 0.001^a^ |
| Sex (Women, n [%]) |  | 40 (80) | 47 (72) | 0.39^b^ |
| Diagnosis (n [%)]) | Idiopathic OA | 23 (46) | 24 (37) | 0.008^b^ |
|  | Post-traumatic OA | 8 (16) | 13 (20) |  |
|  | Post-sprain OA | 7 (14) | 24 (37) |  |
|  | Rheumatoid arthritis | 9 (18) | 3 (5) |  |
|  | Others | 3 (6) | 1 (2) |  |
| Postoperative follow-up (months) |  | 29 (17, 56) | 34 (19, 51) | 0.48^a^ |
| Operative procedure (n [%]) | TNK ankle | 13 (26) | n.a. |  |
|  | TNK ankle + total talar prosthesis | 17 (34) | n.a. |  |
|  | FINE Ankle | 1 (2) | n.a. |  |
|  | TM Ankle | 19 (38) | n.a. |  |
|  | Arthroscopic arthrodesis | n.a. | 60 (92) |  |
|  | Open arthrodesis | n.a. | 5 (8) |  |
| Bilateral operation (n [%]) |  | 2 (4) | 3 (5) | 1.00^b^ |
| Revision operation (n [%]) |  | 1 (2) | 3 (5) | 0.63^b^ |
| Interval between 1^st^ and 2^nd^ FJS (days) |  | 14 (14, 14) | 14 (14, 15) | 0.09^a^ |
| FJS-12 (mean ± standard deviation) |  | 64 ± 20 | 58 ± 26 | 0.20^c^ |
| SAFE-Q | Pain | 87 (77, 100) | 94 (79, 100) | 0.15^a^ |
|  | Physical Functioning | 76 (68, 87) | 77 (64, 89) | 0.79^a^ |
|  | Social Functioning | 88 (74, 100) | 100 (75, 100) | 0.06^a^ |
|  | Shoe-Related | 83 (67, 100) | 83 (67, 96) | 0.27^a^ |
|  | General Health | 93 (80, 100) | 90 (80, 100) | 0.94^a^ |
| EQ-5D-5L | Descriptive system index value | 0.86 (0.74, 0.89) | 0.89 (0.78, 1) | 0.21^a^ |
|  | EQ VAS | 80 (74, 90) | 80 (70, 90) | 0.63^a^ |

The following sentences were added to the table caption.

“^a^Wilcoxon signed-rank test. ^b^Fisher’s exact test. ^c^Student t-test.” (L217 of the manuscript R2)

[Reviewer comment]

2- Page 17, lines 253-259, you were needed to analyze the floor and ceiling effects for FJS-12 scores. You may omit reports findings on the floor and ceiling effects for SAFE-Q scores and EQ-5D-5L. Please clarify it.

[Author response]

We appreciate the reviewer’s comment. Although we believe the information is valuable to highlight the advantage of the FJS-12, this part was deleted according to the reviewer’s comment.

[Author action]

The following sentences were deleted.

“However, most of the SAFE-Q scores, including pain (n = 14 [28%]), social functioning (n = 14 [28%]), shoe-related (n = 14 [28%]), and general health (n = 18 [36%]), and EQ-5D-5L descriptive system index values (n = 11 [22%]), had a ceiling effect in the TAR group. Similarly, in the AA group, SAFE-Q pain (n = 26 [40%]), social functioning (n = 33 [51%]), shoe-related (n = 16 [25%]), and general health (n = 24 [37%]) subscale scores and the EQ-5D-5L descriptive system index values (n = 24 [37%]) had a ceiling effect.” (L253-259 of the manuscript R1)

We also deleted the sentence in the Discussion.

“This contrasts with the SAFE-Q, in which most subscales had ceiling effects.” (L191 of the manuscript R1)

[Reviewer comment]
Discussion
1- This section should also focus on the examination of the Japanese FJS-12 scores known-group validity that was not confirmed. Findings on the factor analysis should also considered for the revised paper.

[Author response]

According to the reviewer’s comment, we mentioned the results of known-group validity and factor analysis in the Discussion.

[Author action]

The following paragraphs were added to the Discussion.

“There was no significant difference in the FJS-12 score between the TAR and AA groups, although the score was slightly higher in the TAR group than in the AA group. This study, therefore, confirmed the construct validity of the questionnaire. Our results were consistent with a recent systematic review that found equality in PROMs after TAR and AA [27]. However, the patients in the TAR group were 8 years older than those in the AA group. Although not the main objective of this study, a comparison of TAA and AA requires further research.” (L283 of the manuscript R2)

“In this study, the principal component analysis showed the two dimensions of the FJS-12, although the eigenvalue for the first factor was six times larger than than that of the second factor (7.06 versus 1.09). In contrast, studies using patients after total knee arthroplasty and anterior cruciate reconstruction reported unidimensionality of the FJS-12 [24, 28]. The difference in the patient background and analysis methods are possible explanations for the discrepancy. Further studies are required to confirm the factor structure of the FJS-12 for patients after TAR and AA.” (L289 of the manuscript R2)

We added the following references.

27. Shih CL, Chen SJ, Huang PJ. Clinical Outcomes of Total Ankle Arthroplasty Versus Ankle Arthrodesis for the Treatment of End-Stage Ankle Arthritis in the Last Decade: a Systematic Review and Meta-analysis. J Foot Ankle Surg. 2020;59:1032-1039. (L428 of the manuscript R2)

28.	Hamilton DF, Loth FL, Giesinger JM, Giesinger K, MacDonald DJ, Patton JT, et al. Validation of the English language Forgotten Joint Score-12 as an outcome measure for total hip and knee arthroplasty in a British population. Bone Joint J. 2017;99-B:218-224. (L431 of the manuscript R2)

The reference numbers in the text and reference list were changed accordingly. (L300, 306, 307, 308, 331, 435, 439, 441, 445, 449, 453, 455 of the manuscript R2)

[Reviewer comment]

2- Page 20, Conclusions, please avoid repetitions of the results and focus instead on the outcomes of the study, and whether you were successful in addressing the gaps as already stated in the Introduction.

[Author response]

According to the reviewer’s comment, the repetitive sentences were deleted. Also, we added a sentence on the clinical relevance.

[Author action]

The following sentences in the conclusion paragraph were deleted.

“…the FJS-12 had sufficient construct validity and internal consistency to assess patients who underwent TAR and AA. It also had acceptable test-retest reliability and measurement errors. No floor or ceiling effects were observed.” (L317-319 of the manuscript R1).

We added the following sentence.

“The FJS-12 can be a useful tool for the postoperative assessment of patients with end-stage ankle arthritis.” (L341 of the manuscript R2)

[Other changes]

We added Yusuke Inaba to the author list.

Yosuke Inaba, Biostatistics Section, Chiba University Hospital Clinical Research Center

Dr. Inaba made a significant contribution to the statistical analysis in this revision, covering the methods, results, and discussion. We would be grateful if you would allow us to add him to authors.

Yosuke Inaba^10^ (L7 of the manuscript R2)

^10^Biostatistics Section, Chiba University Hospital Clinical Research Center (L26 of the manuscript R2)
